# Network Pharmacology Combined with an Experimental Validation Study to Reveal the Effect and Mechanism of *Eucommia ulmoides* Leaf Polysaccharide against Immunomodulation

**DOI:** 10.3390/foods12051062

**Published:** 2023-03-02

**Authors:** Enhui Cui, Pan Tang, Xiaoyan Zhu, Mengyuan Lv, Shuai Wang, Yuhuan Xue, Cixia Li, Shanting Zhao

**Affiliations:** College of Veterinary Medicine, Northwest A & F University, Yangling 712100, China

**Keywords:** *Eucommia ulmoides* leaf, polysaccharide, immunosuppression, pathway

## Abstract

In the present study, the immuno-enhancing effect of *Eucommia ulmoides* leaf polysaccharide (ELP) was investigated in immunosuppressed mice induced by cyclophosphamide (CTX). To evaluate the immune enhancement mechanism of ELP, the immunoregulation effect of ELP was evaluated in vitro and in vivo. ELP is primarily composed of arabinose (26.61%), galacturonic acid (25.1%), galactose (19.35%), rhamnose (16.13%), and a small amount of glucose (12.9%). At 1000~5000 μg·mL^−1^, ELP could significantly enhance the proliferation and the phagocytosis of macrophages in vitro. Additionally, ELP could protect immune organs, reduce pathological damage, and reverse the decrease in the hematological indices. Moreover, ELP significantly increased the phagocytic index, enhanced the ear swelling response, augmented the production of inflammatory cytokines, and markedly up-regulated the expression of IL-1β, IL-6, and TNF-α mRNA levels. Furthermore, ELP improved phosphorylated p38, ERK1/2, and JNK levels, suggesting that MAPKs might be involved in immunomodulatory effects. The results provide a theoretical foundation for exploring the immune modulation function of ELP as a functional food.

## 1. Introduction

The immune system is responsible for recognizing or eliminating ingested pathogens [1]. Immunosuppression affects the body’s ability to prevent diseases and increases its susceptibility to infections following immune system impairment [2]. The immune system is therefore normally maintained to reduce immunosuppression, which has critical implications for protection against disease [3].

The primary goal of immunopotentiators is to enhance the activation of immune responses and resistance to pathogen infection [4]. However, commonly used immunosuppressive agents can cause serious side effects [5]. Chinese herbal medicines have gained considerable attention as natural immunopotentiators in recent years, and some related studies have been conducted [6].

As a famous traditional medical plant, *Eucommia ulmoides* Oliv. contains a rich chemical composition, containing, for example, lignans, polyphenolic acids, iridoids, and flavonoids [7,8]. Its components have anti-oxidative, anti-bacterial, anti-tumor and anti-inflammatory effects, and also delay aging, improve immunity, and lower blood pressure [9,10]. *Eucommia ulmoides* Oliv., a functional health food, has been used to treat hypo-immunity, osteoporosis, cardiovascular disease, rheumatoid arthritis, and the menopause [11,12]. Modern pharmacological studies have proven that the active ingredients and efficacy of *Eucommia ulmoides* leaves are similar to those of *Eucommia ulmoides* Oliv. Furthermore, previous studies have shown that *Eucommia ulmoides* leaf polysaccharide (ELP) has the potential to improve immune responses [13]. At the same time, it has a broad spectrum of biological activities, of which antioxidant activity is an important one [14]. However, studies have largely focused on the biological activity of *Eucommia ulmoides* bark, and the leaves have been ignored. More importantly, as far as we know, there are as yet no reports on ELP modulation of CTX-induced immunosuppression.

Therefore, to investigate the immunomodulatory effects of ELP, crude polysaccharides with good properties and purity were initially extracted from the *Eucommia ulmoides* leaf. Then, a CTX-induced immunosuppression mice model was established and intervened with ELP and saline. Network pharmacology was applied to identify the potential therapeutic targets and important signaling pathways associated with immune dysregulation, and further experiments were performed to validate the effects and the mechanism of ELP in CTX-induced immunosuppression.

## 2. Materials and Methods

### 2.1. Preparation of ELP

The leaves of *Eucommia ulmoides* were collected on the campus of Northwest Agricultural and Forestry University of China. *Eucommia ulmoides* leaves were degreased with 95% alcohol, and extracted three times with distilled water (20 L) at 80 °C. Extracts of *Eucommia ulmoides* leaves were then concentrated to the desired volume. About 4 times the amount of ethanol solution was added to the concentrated liquor and left to set overnight at 4 °C. After collecting the precipitate, it was dialyzed against distilled water. The dialysis solution was freeze-dried after rotary evaporation under vacuum to obtain the crude *Eucommia ulmoides* leaf polysaccharide. Then, *Eucommia ulmoides* leaf polysaccharide was purified with a DEAE-52 cellulose column and eluted with distilled water and gradient NaCl solutions of 0.05, 0.1, 0.2, and 0.5 mol/L. The eluted fractions were pooled and concentrated, and the concentration of neutral saccharides was determined using the phenol-sulfuric acid method. The polysaccharide fractions were again dialyzed against water for 3 days and lyophilized [15].

### 2.2. Physiochemical Analysis of Eucommia ulmoides Leaf Polysaccharide

*Eucommia ulmoides* leaf polysaccharides were analyzed by high-performance liquid chromatography (HPLC). The polysaccharide was hydrolyzed with 2 M TFA at 110 °C for 4 h, and then PMP reagent was added for derivatization. A Cosmosil 5C18-PAQ column (4.6 × 150 mm) was used, and the detection wavelength was fixed at 254 nm for the analysis. The HPLC mobile phase consisted of a mixture of 80% 0.05 M KH_2_PO_4_ (pH = 6.9) and 20% acetonitrile. The injection volume was 20 μL and the flow rate was 1.0 mL/min [16].

### 2.3. Phagocytic Activity of Macrophages

Neutral red uptake was used to evaluate the phagocytic activity of macrophages [17]. Due to the endocytosis of macrophages, neutral red dye can aggregate into macrophages to form red dot aggregates. BALB/c mice were injected with 6% starch-broth medium (1.0 mL, i.p.) 3 days before the trial ended. Primary macrophages were harvested by flushing with 20 mL of PBS at the end of the experiment. The cells were collected and washed with PBS twice after centrifugation at 2000 rpm for 10 min. Cells were resuspended in RPMI to a density of 1 × 10^6^ cells/mL, seeded in culture plates, and incubated overnight at 37 °C with 5% CO_2_ in the incubator. The adherent cells were macrophages. Macrophages were treated in the presence of ELP at a concentration of 1000~5000 μg/mL for 24 h, respectively. The control group was incubated without ELP. Then, 100 μL neutral red solutions (0.075%, *w*/*w*) were added into the medium and the cells were incubated for 4 h. After removing the supernatant, the cells were rinsed twice with PBS to discard the unphagocytized neutral red, followed by addition of DMSO (100 μL/well) lyse macrophages. The optical density was detected with a microplate reader at an absorbance of 570 nm.

### 2.4. Experimental Design and Drug Administration

A total of 60 six-to-eight-week-old (weighing 18–22 g) male BALB/c mice were purchased from the Laboratory Animals Center of Xi’an Jiaotong University (Shaanxi, China). All mice were maintained in a 12 h: 12 h on/off photoperiod (light from 9:00–21:00), with food and water freely available. Before experiments, mice were housed under the same conditions for 7 days to acclimatize. Each cage contained four mice. All animal experiments were conducted according to the guidelines for Care and Use of Laboratory Animals of Northwest A&F University, and protocols were approved by the Animal Care and Use Commission of the College of Veterinary Medicine, Northwest A&F University (certificate no. SCXK [SHAAN] 2021-003).

Cyclophosphamide (CTX, Solabio, Beijing, China) was used to induce an immunosuppressive model. After an acclimation period, mice were randomly divided into three groups, each group contained 20 mice and was administered intraperitoneally either normal saline (normal control group), 70 mg/kg CTX (CTX-induced immunosuppression model group), or 70 mg/kg CTX+ELP (ELP group). Except for the untreated mice in the control group, the mice in the other groups were intraperitoneally injected with CTX (70 mg/kg) solution for 7 days (day 15 to 21) to establish the immunosuppressive model. ELP was administered once daily at a 200 mg/kg dosage. The dose of CTX (70 mg/kg) was based on [18]. The dosage of ELP (200 mg/kg) was based on [19]. On day 21, animals were euthanized by CO_2_ asphyxiation after an overnight fast.

### 2.5. Determination of Immune Organs Indices

After the mice were sacrificed, the thymus and spleen were weighed. The immune organ index was calculated by the following formula:

Thymus or spleen index = weight of thymus or spleen (mg)/body weight (g).(1)

### 2.6. Histological Examinations of the Liver and Spleen

A histopathological examination was undertaken to evaluate the morphological changes in the liver and spleen. Approximately 5 mm by 5 mm tissue blocks were fixed for 24 h in 4% paraformaldehyde in 0.1 M PB (pH = 7.40). After dehydration in graded ethanol and dimethylbenzene, the fixed tissues were embedded in paraffin. Then, 4µm-thick sections were sliced with a microtome, mounted on glass slides, and stained with hematoxylin-eosin (H&E) according to a standard staining protocol for microscopic examination.

### 2.7. Determination of Cytokines in Serum

Concentrations of IL-2/IL-4/IFN-γ/TNF-α in serum samples were examined by mouse cytokines ELISA kits (Enzyme-linked Biotechnology Co., Ltd., Shanghai, China) according to the manufacturer’s instructions.

### 2.8. Peripheral Blood Leukocyte and Lymphocyte Counts

Blood was collected by retro-orbital bleeding from mice under anesthesia. Peripheral hemogram analyses were performed with the auto hematology analyzer BC-2800vet (Wuhan Servicebio Technology Co., Ltd., Wuhan, China).

### 2.9. Determination of the Phagocytic Index

The phagocytic capacity of blood macrophages was examined by a carbon particle clearance test [20], and each mouse was injected with a 4-fold dilution of 100 μL Indian ink via the tail vein. Blood was collected at 2 min (tA) and 10 min (tB) after injection from the orbital sinus, followed by mixing with 2 mL 0.1% Na_2_CO_3_, respectively. Then, the optical density was measured at 600 nm (ODA and ODB). The phagocytic index (α) was calculated using the formula:

K = (lgODA − lgODB)/(tB − tA)(2)

Phagocytic index, α = K(1/3) × WB/(WL + WS)(3)

where WB denotes the body weight and WL and WS are the liver weight and spleen weight, respectively.

### 2.10. Delayed-Type Hypersensitivity (DTH) Test

The cell-immunity function was evaluated by the DTH reaction [21]. For sensitization, 20 μL DNFB (5% dissolved in 1:1 acetone/olive oil) was applied to the shaved abdomens on day 0 and day 1. Five days later, animals were rechallenged by applying 10 μL of 5% DNFB on the dorsal side of the right ear. Furthermore, ear samples were dissected and weighed. After that, the DTH response was assessed by the difference in the weight between the right and left ears.

### 2.11. qRT-PCR Analysis

Total RNA was extracted from the mouse spleens with a SteadyPure Universal RNA Extraction kit and reverse transcribed using an Evo M-MLV RT Mix Kit following the manufacturer’s instructions. The RT reactions were then carried out at 37 °C for 15 min followed by heating at 85 °C for 5 s to denature the RT. qRT-PCR was performed using a SYBR^®^ Green Premix Pro Taq HS qPCR kit. All PCR-related kits were purchased from Accurate Biotechnology Co., Ltd. (Hunan, China). The forward (F) and reverse (R) primer sequence is shown in Table 1. The reaction conditions of the RT-PCR were 95 °C for 30 s, followed by 95 °C for 5 s and 60 °C for 30 s for 40 cycles. The relative gene expression was determined using the 2−ΔΔCT method.

### 2.12. Western Blot Assay

Mouse spleens were collected and dissolved in RIPA buffer (pH = 7.4) with a protease inhibitor (P1400, Solarbio, Beijing, China). After determining the protein concentration using the BCA Protein Assay kit (Solarbio), protein samples were boiled for 5 min at 100 °C, followed by separation on 10% SDS-PAGE gel, and were then transferred onto polyvinylidene difluoride membranes (Millipore, Darmstadt, Germany). After blocking with 5% milk powder dissolved in TBST at room temperature for 2 h, the membranes were incubated with the relevant primary antibody followed by an appropriate secondary antibody. The main primary antibodies were p38 MAPK (1:1000), JNK (1:1000), ERK (1:1000), p-JNK (1:1000), p-ERK (1:1000), and p-p38 MAPK (1:1000). β-tubulin was used as an internal control. Exposed films were scanned and protein bands were quantified using the Q9 Alliance software (UVItec, Cambridge, UK).

### 2.13. Network Pharmacology Analysis

The target of ELP was predicted using the TCMSP database and previous studies. “Immune dysregulation” associated targets were determined on the website https://www.omim.org/ (OMIM database) and https://www.genecards.org/ (Gene-Cards database), accessed on 10 February 2022. The gene name of the target protein was queried in the Uniprot database.

To obtain overlapping targets of compounds and disease, we first cross-contrasted ELP-related targets with immune dysregulation-related targets. Then, the interaction target genes were mapped in the STRING database to visualize and analyze the protein–protein interaction (PPI) network. The network parameters of nodes in the PPI network were generated using the CytoNCA plugin in Cytoscape 3.8.2. The key targets were selected based on the network parameters. A Kyoto Encyclopedia of Genes and Genomes (KEGG) pathway enrichment analysis was performed using R software (version 3.6.3).

### 2.14. Statistical Analysis

All data from the study were analyzed using IBM^®^ SPSS statistics version 26 and expressed as means and standard error (mean ± SEM). Differences between groups were compared using one-way ANOVA followed by the Tukey HSD test. In vitro experiments were performed independently at least 3 times and in vivo experiments were performed with the number of mice as shown in the figure legend. A *p* < 0.05 was considered statistically significant.

## 3. Results

### 3.1. Physicochemical Properties of Eucommia ulmoides Leaf Polysaccharide

**Figure 1:**
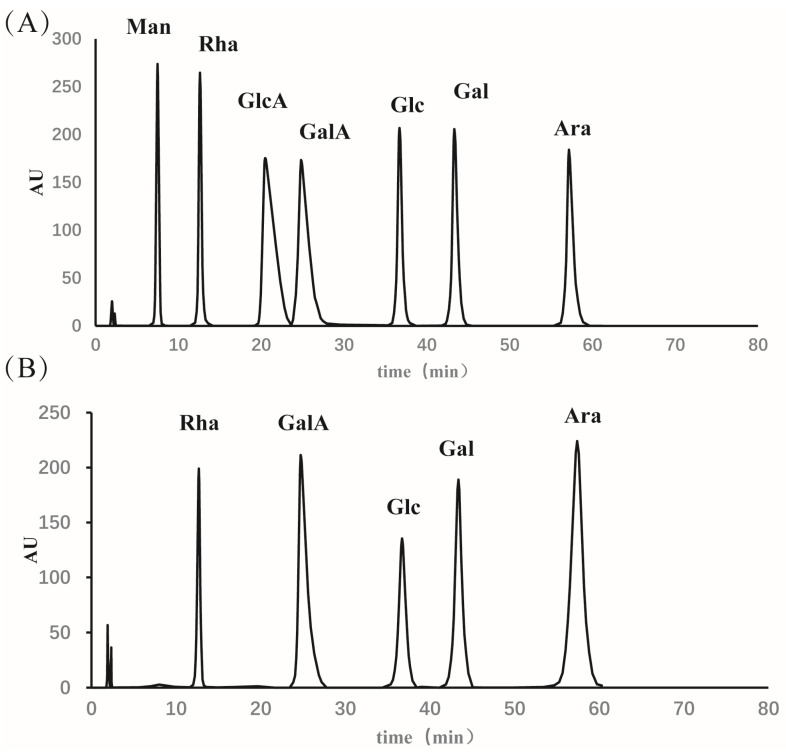
Monosaccharide composition analysis by HPLC. (**A**) Monosaccharide Standard and (**B**) ELP. Ara: arabinose, GalA: galacturonic acid, Gal: galactose, Rha: rhamnose, Glc: glucose.

The neutral sugar, uronic acid, and protein contents were 68.5%, 21.67%, and 0.98%, respectively. As shown in Figure 1, ELP was primarily composed of arabinose (26.61%), galacturonic acid (25.1%), galactose (19.35%), rhamnose (16.13%), and a small amount of glucose (12.9%).

### 3.2. Effects of ELP on Immunoregulation In Vitro

#### 3.2.1. Effect of ELP in Macrophage Proliferation of Mice

**Figure 2:**
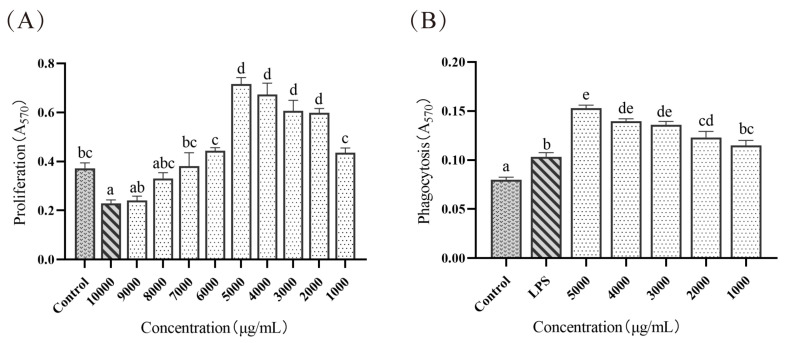
(**A**) The effect of ELP on macrophage proliferation of mice. (**B**) The effect of ELP on macrophage phagocytosis of mice. Different superscript letters are used to represent significant differences: bars with the same superscript letter indicate no statistical differences (n.s), while bars with different letters indicate pairwise *p* < 0.05. Six mice/group were used for this analysis.

Cells were treated with different concentrations of ELP. As shown in Figure 2A, the A570 value of cells treated with ELP at a concentration of 10 000 μg/mL was remarkably lower than that of the control group (0.2284 ± 0.0147 vs. 0.3721 ± 0.022, *p* = 0.032), indicating that ELP inhibited the growth of cells at this concentration. The A570 value of cells treated with ELP at 6000~9000 μg/mL concentrations was not significant (*p* = 0.843, *p* = 1.000, *p* = 0.997, *p* = 0.073), which suggested that ELP had no significant impact on cell viability at these concentrations. The cell viability increased as ELP concentration increased (1000~5000 μg/mL (0.436 ± 0.019, 0.599 ± 0.018, 0.606 ± 0.043, 0.674 ± 0.045, 0.717 ± 0.026)) in a dose-dependent manner.

#### 3.2.2. Effect of ELP on Macrophage Phagocytosis in Mice

The effect of ELP on macrophage phagocytosis is illustrated in Figure 2B. The A570 values in cells treated with ELP at 1000~5000 μg/mL were significantly higher than that in the control group (*p* < 0.001, *p* < 0.001, *p* < 0.001, *p* < 0.001, *p* < 0.001), indicating that ELP could promote macrophage phagocytosis.

### 3.3. Effects of ELP on Immunoregulation In Vivo

#### 3.3.1. Effects of ELP on Immune Organ Indexes

**Figure 3:**
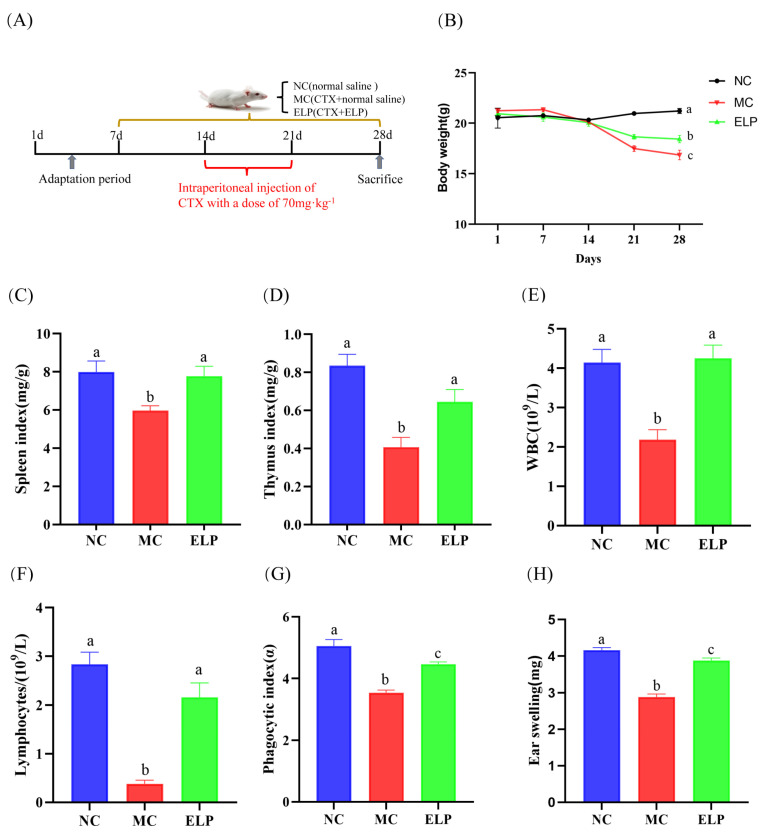
ELP administration inhibited CTX-induced impairment of body weight and the immune system. (**A**) Schematic diagram of the experimental procedures. Effect of ELP on changes in body weight (**B**), spleen index (**C**), thymus index (**D**), WBCs (**E**), lymphocytes (**F**), phagocytic index (**G**), and DTH reaction (**H**) in mice treated with CTX. NC: normal control group, MC: CTX-induced immunosuppression model group, ELP: ELP group, and the same as below. Data are represented as means ± SEM. Different superscript letters are used to represent significant differences: bars with the same superscript letter indicate no statistical differences (n.s), while bars with different letters indicate pairwise *p* < 0.05. Six mice/group were used for this analysis.

Figure 3A shows the schematic of the experimental protocols. Body weight is a basic indicator of the general condition of mice, while the thymus and spleen are major organs of the immune system [22]. The changes in and a comparison of body weight during experiments are shown in Figure 3B. After seven consecutive days of CTX treatment, the mice began to lose weight. There were significant differences between the control (20.975 ± 0.192) group and CTX-treated groups (CTX:17.487 ± 0.291 and ELP:18.663 ± 0.234). On day 28, the mice in the ELP group had a higher body weight than the MC group (18.425 ± 0.364 vs. 16.850 ± 0.476, *p* = 0.020).

The immune organ index was dramatically lower in the MC group (spleen index: 5.982 ± 0.251 and thymus index: 0.407 ± 0.052) than that in the NC group (spleen index: 7.994 ± 0.581 and thymus index: 0.835 ± 0.060), whereas the administration of ELP significantly increased the immune organ index (spleen index: 7.768 ± 0.528 and thymus index: 0.645 ± 0.065) (Figure 3C,D).

#### 3.3.2. Peripheral Blood White Blood Cells (WBCs) and Lymphocytes

The peripheral blood index is a common clinical measure reflecting the body’s immunity and infection status [23]. As seen in Figure 3E,F, compared with those in the control group, the densities of WBCs (2.183 ± 0.256, *p* < 0.001) and lymphocytes in peripheral blood (0.383 ± 0.070, *p* < 0.001) decreased notably in the model group with CTX administration, whereas the densities of WBCs and lymphocytes increased significantly in the ELP group (4.250 ± 0.339, *p* = 0.001 and 2.167 ± 0.291, *p* < 0.001). The results revealed that ELP could improve the immunosuppression induced by CTX.

#### 3.3.3. Phagocytic Index

The monocyte/macrophage phagocytic function was reflected by the carbon particle clearance test [20]. As shown in Figure 3G, the phagocytic index (α) was noticeably lower in the model group than the control group (3.546 ± 0.086 vs. 5.051 ± 0.216, *p* < 0.001). However, the phagocytic index was substantially higher in the ELP group than the model group, suggesting that ELP could enhance the phagocytic activity (4.460 ± 0.079 vs. 3.546 ± 0.086, *p* < 0.001).

#### 3.3.4. DTH Reaction

In vivo immune responses were evaluated using the DTH response [24]. The earlap swelling of mice was drastically alleviated in the MC group when compared to that in the NC group (2.885 ± 0.082 vs. 4.164 ± 0.07, *p* < 0.001), indicating that the model was successfully established (Figure 3H). The swelling of the ear was more severe in the ELP group than in the model group (3.883 ± 0.067 vs. 2.8850 ± 0.082, *p* < 0.001), suggesting that ELP could exert specific immunity by improving DTH reaction.

#### 3.3.5. Histological Analysis of Spleen and Liver

**Figure 4:**
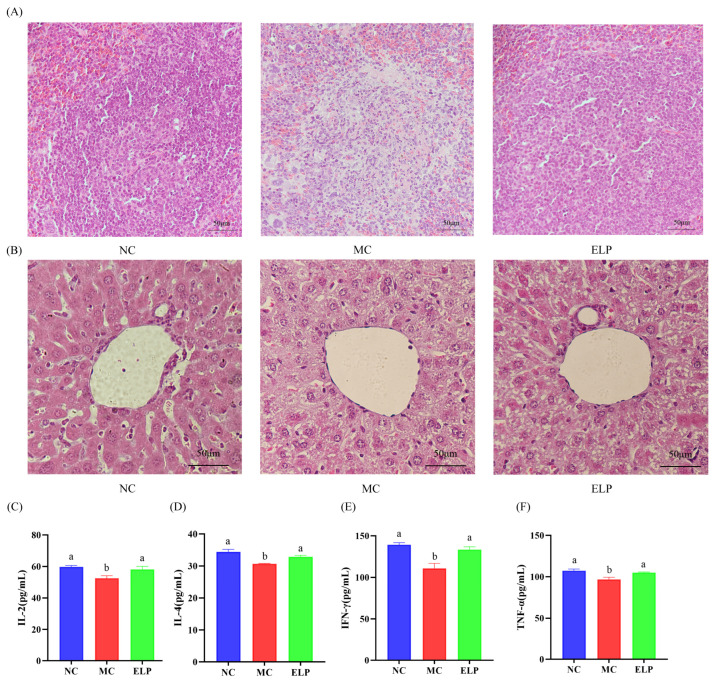
Detailed histological analyses of the spleen (**A**) and liver (**B**) in BALB/c mice. Scale bar: 50 μm. Effect of ELP on cytokines of IL-2 (**C**), IL-4 (**D**), IFN-γ (**E**), and TNF-α (**F**)**.** Data are represented as means ± SEM. Different superscript letters are used to represent significant differences: bars with the same superscript letter indicate no statistical differences (n.s), while bars with different letters indicate pairwise *p* < 0.05. Six mice/group were used for this analysis.

To evaluate the CTX-induced organ damage, the H&E-stained sections of the spleen and liver were analyzed. The H&E staining of the spleen is shown in Figure 4A. The splenic white and red marrow in the NC group were demarcated and had fewer intracellular vacuoles. However, cells in the MC group were arranged irregularly. Furthermore, the boundaries between the red pulp and the white pulp in the spleen were unclear, and the necrosis area was obvious compared with the NC group. After treatment with ELP, the spleen showed fewer necrotic areas and vacuoles and cells were regularly arranged. These results suggest that ELP could effectively improve the organ damage caused by CTX.

As shown in Figure 4B, in the liver sections, the hepatic lobules in the control group were clear and intact, the liver cells were uniform, the nucleus was visible, and the hepatic cords were arranged in a radial form. In contrast, hepatocytes in CTX-treated mice were arranged loosely and massively vacuolized. However, ELP could effectively ameliorate liver injury and dramatically reduce hepatocyte vacuoles.

#### 3.3.6. Effects of ELP on Serum Cytokine Levels

The levels of IL-2, IL-4, IFN-γ, and TNF-α were substantially lower in the MC group (52.505 ± 1.677, 30.672 ± 0.172, 110.812 ± 6.074, and 96.8066 ± 2.608) than those in the NC group (59.847 ± 0.858, 34.440 ± 0.777, 139.363 ± 2.452, and 107.467 ± 1.934), while the cytokine levels were significantly higher in the ELP group (58.215 ± 1.857, 32.899 ± 0.464, 133.408 ± 3.389, and 104.965 ± 0.873) than in the MC group (*p* < 0.05) (Figure 4C–F). ELP can reverse immunosuppression and boost immunity by enhancing cytokine production.

#### 3.3.7. KEGG Pathway Enrichment

**Figure 5:**
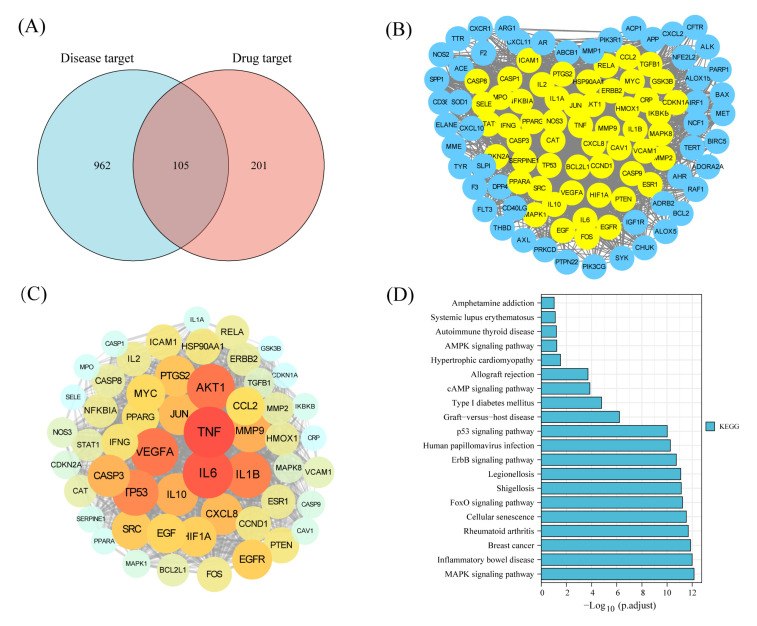
Network pharmacology prediction of ELP on immune dysregulation. (**A**) The intersection of ELP targets and disease targets. (**B**) Results of PPI analysis for potential targets of ELP. (**C**) A total of 21 core targets of ELP act on immune dysregulation. (**D**) KEGG enrichment analysis for the potential pathways of ELP.

To systematically explore the mechanism of protective effect of ELP on immunosuppression, the potential targets of ELP in immune dysregulation were analyzed by applying network pharmacology. The parameters of ELP were shown in Table 2. There were 105 intersecting targets between ELP and immune dysregulation (Figure 5A). A PPI network analysis of 105 cross-targets yielded 21 core target genes of ELP acting on immune dysregulation (Figure 5B,C). As shown in Figure 5D, the KEGG pathway analysis shows that the enrichment of genes is associated with MAPK pathways. These results suggested that ELP could exert pharmacological effects through multi-targets and multi-pathways.

#### 3.3.8. Effects of ELP on Gene Expression

**Figure 6:**
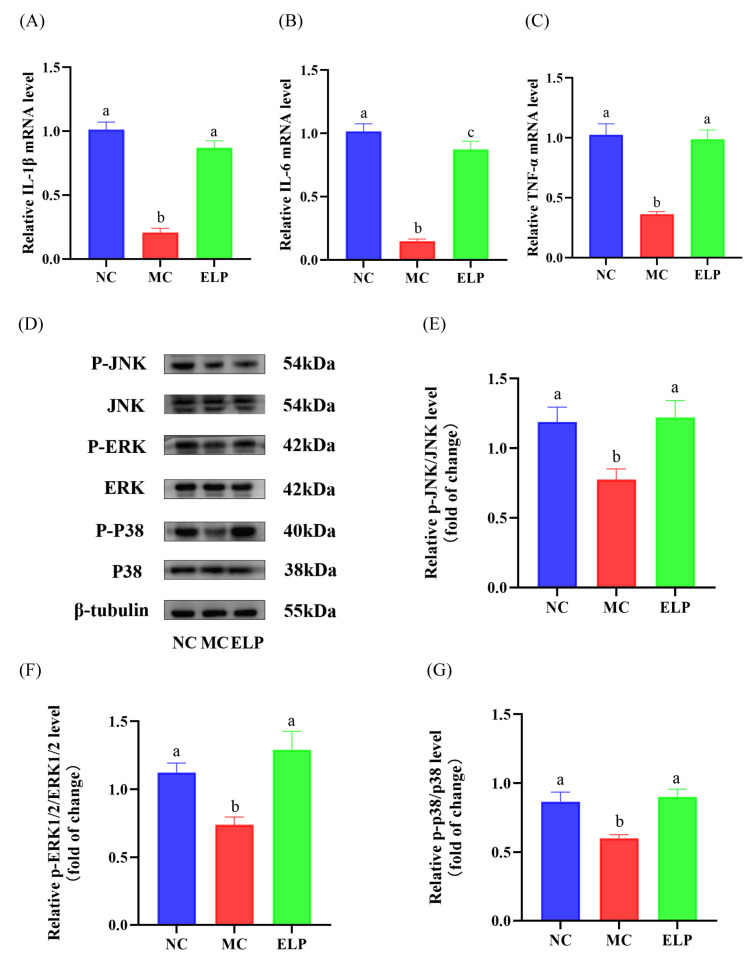
ELP inhibited the increase in inflammatory mediators induced by CTX (**A**–**C**). Comparison of the mRNA expression levels of IL-1β (**A**), IL-6 (**B**), and TNF-α (**C**) in different groups. ELP up-regulated p-JNK/JNK, p-ERK/ERK, and p-p38/p38 in the CTX-treated mice (**D**)**.** Western blots of p-JNK, JNK, p-ERK, ERK, p-p38, and p38 in the spleens. A quantification of densitometric analyses was performed by measuring the expression ratios of p-JNK/JNK (**E**), p-ERK/ERK (**F**), and p-p38/p38 (**G**) in the spleens (n = 8 per group). ImageJ software was used to quantify immunoblot bands. Data are represented as means ± SEM, and a one-way ANOVA test was used to evaluate the statistical significance. Different superscript letters are used to represent significant differences: bars with the same superscript letter indicate no statistical differences (n.s), while bars with different letters indicate pairwise *p* < 0.05. IL-1β: interleukin-1 beta; IL-6: interleukin-6; TNF-α: tumor necrosis factor-α. Six mice/group were used for this analysis.

A total of 21 core targets of *Eucommia ulmoides* leaf acting on immune dysregulation were selected (Figure 5C). The expression of IL-1β, IL-6, and TNF-α at mRNA levels in the spleen was evaluated by qRT-PCR (Figure 6A–C). They were significantly lower in the CTX group than those in the normal group (IL-1β: *p* < 0.001, IL-6: *p* < 0.001, and TNF-α: *p* < 0.001). In addition, the mRNA levels of IL-1β, IL-6, and TNF-α in the ELP group were significantly higher than those in the MC group (IL-1β: *p* < 0.001, IL-6: *p* < 0.001, and TNF-α: *p* < 0.001). These results indicate that ELP may ameliorate spleen dysfunction caused by CTX.

#### 3.3.9. Western Blot Analysis

A network pharmacology enrichment analysis confirmed the alterations in the MAPK pathway in immunosuppression. The protein expression of p-JNK, p-ERK, and p-p38 was confirmed by Western blotting (Figure 6D). Western blotting results revealed that the significantly decreased expression of p-JNK, p-ERK, and p-p38 in the CTX group (p-JNK:0.777 ± 0.076, p-ERK:0.740 ± 0.057, and p-p38:0.601 ± 0.027) was restored by ELP intervention (p-JNK:1.220 ± 0.123, p-ERK:1.292 ± 0.135, and p-p38:0.902 ± 0.056; Figure 6E–G). In summary, ELP may exert its immunoregulatory effects in CTX-treated mice by activating the MAPK signaling pathways.

## 4. Discussion

CTX, a nitrogen mustard alkylating agent, is widely used to treat malignant diseases [25]. Nevertheless, CTX can cause DNA damage and result in immunosuppression. Modulation of the immune response to mitigate disease has attracted considerable interest over the years. Polysaccharides obtained from plants have gained interest for their potential immunomodulatory activities. As a “drug homologous food” in China, Eucommia leaf is rich in many active ingredients. However, little research has been reported on the polysaccharide composition and immune enhancing activity of *Eucommia ulmoides* leaf. Therefore, we used CTX to establish an immunodeficiency model in mice [25]. In this study, we found that CTX decreased mice body weight and immune organ indices, lowered the macrophage phagocytic activity, and restricted the DTH reactions and lymphocyte density in peripheral blood. These results suggest that the CTX-induced immunosuppression model is successfully established. Intervention with polysaccharides from Eucommia globulus leaves improved the immune activity in immunosuppressed mice.

Immune suppression is manifested at the cellular level in terms of altered macrophage activity, suppressed proliferation, and decreased phagocytosis. The plant polysaccharides regulate immune function mainly by affecting macrophage proliferation and phagocytic activity. Therefore, macrophage proliferation is often used as one of the indicators to evaluate macrophage function. The results of this trial showed that cell viability increased with an increase in ELP concentration (1000–5000 μg/mL) in a dose-dependent manner, indicating that a certain concentration of ELP could improve the proliferation of mouse peritoneal macrophages. Meanwhile, the phagocytic capacity of macrophages is one of the important indicators of the non-specific immune function of the body. The A570 values in vitro in ELP at 1000~5000 μg/mL were highly significantly higher than those in the control group (*p* < 0.01) and also significantly higher than those in the LPS group, suggesting that a certain concentration of ELP also significantly enhanced the phagocytosis of macrophages. These results showed that ELP promoted macrophage proliferation, leading to an increase in the number of macrophages, which in turn enhanced macrophage phagocytosis.

Body weight is an indicator of the overall condition of mice. Compared with the control group, the mice in the MC group showed a significant reduction in body weight. When mice were treated with ELP, their body weight was significantly restored, indicating that ELP could attenuate the weight loss caused by CTX. The spleen and thymus are the two most important immune organs in the body’s immune system that maintain immune homeostasis [26,27]. The thymus is a place for T-cell differentiation and maturation and has direct effects on the level of cellular immunity and indirect effects on the immunity of the humor. The spleen is an important peripheral immune organ that differentiates into a large number of T and B lymphocytes to participate in immunity after stimulation. The organ index is a preliminary indicator of organism immunity. In the present study, ELP treatment can remarkably increase the immune organ indexes in CTX-treated mice. Similarly, the immune status of mice can also be determined by the histomorphology of the liver and spleen. Hepatocytes in the CTX group were in a disorganized state, and the demarcation line between splenic white marrow and splenic red marrow was not obvious. However, histological examinations showed that ELP can effectively alleviate spleen and liver injury, indicating that ELP can exert its immunomodulatory role by influencing the immune organs.

Non-specific immunity not only responds rapidly to various pathogenic microorganisms but also plays an important role in the initiation of specific immunity. The carbon particle profile is a common method for assessing the non-specific immune function of the immune system. As a component of the immune system, macrophages engulf bacteria and have antineoplastic properties. They play a fundamental role in activating the innate immune response [28]. The macrophage phagocytic index was measured to reflect the cellular immune effect of ELP on immunosuppressed mice in the present study. The carbon particle clearance method is now commonly used to measure the phagocytic index of phagocytes. Consistent with previous reports, our results demonstrate that CTX can suppress macrophage activity [29]. Interestingly, ELP can reverse the declined phagocytosis and have an immune protective effect in immunosuppressed mice. These results confirm that ELP has the effect of promoting the non-specific immune function of the body.

As a class IV hypersensitivity response, DTH is an important type of cell-mediated pathological response that plays a key role in the assessment of T cell-mediated immune responses. We induced an ear DTH reaction with DNFB, and the swelling of the ear increased after treatment with ELP. This implies that ELP reverses CTX’s inhibitory effect on the DTH reaction, indicating that ELP may contribute to the cellular immune response.

The preferred method for diagnosing diseases is via routine blood tests [30]. WBCs are composed of lymphocytes, monocytes, and granulocytes. The density of leucocyte in peripheral blood is the most widely used potential immune biomarker of infection [31]. Additionally, CTX is associated with the side effect of inhibiting bone marrow hematopoietic function and reducing the number of WBCs and lymphocytes [32,33]. Our results demonstrate that ELP can restore hematopoiesis in CTX-induced immunosuppressed mice.

Cytokines, soluble mediators of immune homeostasis, have multiple functions and display pleiotropic effects [34]. Cytokines are useful for disease prevention and treatment. Increased IL-2, IL-4, TNF-α, and IFN-γ levels can enhance macrophage activity and boost immunity. IL-2, a polypeptide cytokine, can enhance the immune functions of various immune cells. It has the functions of maintaining cell proliferation; promoting B cell proliferation, differentiation, and antibody secretion; and inducing differentiation and effects on various killer cells such as CTL and NK. The secretion of IFN-γ and IL-4 are related to the Th1 and Th2 cell responses, respectively [35]. IFN-γ can activate macrophages to kill pathogenic microorganisms, promote B cell differentiation to produce antibodies, activate NK cell killing potential, etc. TNF-α, a proinflammatory cytokine, is involved in immune modulation [36]. In the present study, the production of IL-2, IL-4, TNF-α, and IFN-γ in serum was significantly reduced in CTX-treated mice, indicating that cyclophosphamide is an immunosuppressive agent. However, the levels of IL-2, IL-4, TNF-α, and IFN-γ in serum were higher in the ELP group than in the CTX group, indicating that ELP can improve immunity functions by improving the levels of immune cytokines. As the two main subpopulations of CD4+ T helper cells, Th1 and Th2 cells can maintain a balanced state and secrete different types of cytokines. IL-2 leads to a Th1 cell response, while IL-4 leads to a Th2 cell response. Our results suggest that ELP may improve the immune function by regulating the Th1/Th2 balance in immunosuppressed mice.

This paper proposes a systematic pharmacology strategy to investigate the immunomodulatory mechanisms of ELP at the molecular pathway and network levels. Systematic pharmacology is a strategy for predicting therapeutic mechanisms that can effectively provide us with a research direction. Network pharmacology research combines systems biology and computer technology, thus leading to an understanding of the material basis and biological mechanisms of drugs. To screen possible target genes and pathways of ELP, this study conducted a network pharmacology prediction using TCMSP, OMIM, GeneCard, Uniprot, and other databases. The results showed that ELP has complex targets, involving multiple signaling pathways such as the MAPK signaling pathway, inflammatory bowel disease, breast cancer, etc.

A total of 21 core genes were obtained based on the PPI network. Immunoregulatory cytokines are small proteins/polypeptides mediating immunity and markers of inflammatory changes. IL-1β, IL-6, and TNF-α are the major core molecules, which may play pivotal roles in immunomodulatory effects. These three molecules are prototypic proinflammatory cytokines and participate in inflammatory reactions and tissue maintenance [37]. Therefore, the mRNA levels of IL-1β, IL-6, and TNF-α were measured and compared in different groups. The data showed that the expressions of IL-1β, IL-6, and TNF-α mRNA are significantly decreased in CTX-administrated mice owing to its immunosuppressive effects, while they are dramatically enhanced by ELP. Therefore, ELP may exert immunomodulatory effects through these targets. A pathway-based enrichment analysis showed that ELP exhibits immunomodulatory effects by targeting MAPK signaling pathways. MAPK pathways widely participate in the induction of proinflammatory cytokine and immune-regulatory proteins [38]. ERK, JNK, and p38 protein kinases belong to the MAPK family [39]. Therefore, we further investigated the MAPK pathway-mediated immune response. Our results demonstrated that ELP supplementation significantly up-regulates p-p38, p-JNK, and p-ERK1/2 protein expression, revealing that ELP may play an immunomodulatory role by activating MAPK signaling pathways.

## 5. Conclusions

In the present study, ELP has been shown to possess good immune enhancement both in vitro and in vivo to alleviate immunosuppression. ELP is primarily composed of arabinose, galacturonic acid, galactose, rhamnose, and a small amount of glucose. Moreover, ELP could significantly enhance the proliferation and the phagocytosis of macrophages in vitro. Based on the network pharmacology, IL-1β and IL-6, as well as TNF-α, were considered as the key targets, and ELP may protect against immunosuppression in cyclophosphamide-treated mice via activating the MAPK signaling pathways. In vivo experiments showed that ELP can restore the body weight of mice, protect immune organs, and significantly improve the serum levels of IL-2, IL-4, TNF-α, and IFN-γ. ELP also up-regulated the mRNA (IL-β, IL-6, and TNF-α) and protein levels of p-JNK, JNK, p-ERK, ERK, p-p38 MAPK, and p38 MAPK. These results provide a theoretical basis for exploring the modulating immune function of ELP as a functional food.

## Figures and Tables

**Table 1:** Primer sequence of polymerase chain reaction (RT-qPCR).

Target Gene	Forward Primer	Reverse Primer
TNF-α	AGTCCGGGCAGGTCTACTTT	GTCACTGTCCCAGCATCTTGT
IL-1β	TGACGGACCCCAAAAGATGA	TCTCCACAGCCACAATGAGT
IL-6	CACTTCACAAGTCGGAGGCT	CTGCAAGTGCATCATCGTTGT
GAPDH	AGGTTGTCTCCTGCGACTGCA	GTGGTCCAGGGTTTCTTACTCC

**Table 2:** Parameters of *ELP*.

NO.	Compound	OB/%	DL
MOL000382	Arabinose	1.87	0.02
MOL011643	Galacturonic acid	40.39	0.06
MOL000814	Galactose	47.94	0.04
MOL000424	Rhamnose	50.50	0.04
MOL000734	Glucose	24.44	0.03

## Data Availability

DATA is contained within the article.
